# A single-cell transcriptomic study of heterogeneity in human embryonic tanycytes

**DOI:** 10.1038/s41598-024-66044-7

**Published:** 2024-07-04

**Authors:** Yiguang Bai, Qiaoling Chen, Yuan Li

**Affiliations:** 1 Department of Orthopaedics, The Second Clinical Institute of North Sichuan Medical College Nanchong, Nanchong Central Hospital, Nanchong, Sichuan China; 2 Department of Oncology, The Second Clinical Institute of North Sichuan Medical College Nanchong, Nanchong Central Hospital, Nanchong, Sichuan China; 3 Nanchong Hospital of Beijing Anzhen Hospital Capital Medical University Sichuan, Beijing, China; 4 National Bioinformatics Infrastructure Sweden (NBIS), Science for Life Laboratory, Lund University, 223 87 Lund, Sweden; 5 Department of Immunotechnology, Lund University, Medicon Village, 22387 Lund, Sweden; 6 Human Neural Developmental Biology; BMC B11, Department of Experimental Medical Science Lund, Stem Cell Centre, Lund University, 22184 Lund, Sweden; 7 Cell, Tissue & Organ Engineering Laboratory; BMC B11, Department of Clinical Sciences Lund, Stem Cell Centre, Lund University, 22184 Lund, Sweden

**Keywords:** Human embryonic tanycyte, Single cell transcriptomics, Heterogeneity, Regulon, Transcription factor, Intercellular communication, Cell–cell communication, Developmental trajectory, Computational biology and bioinformatics, Developmental biology, Neuroscience

## Abstract

Disruptions in energy homeostasis can lead to diseases like obesity and diabetes, affecting millions of people each year. Tanycytes, the adult stem cells in the hypothalamus, play crucial roles in assisting hypothalamic neurons in maintaining energy balance. Although tanycytes have been extensively studied in rodents, our understanding of human tanycytes remains limited. In this study, we utilized single-cell transcriptomics data to explore the heterogeneity of human embryonic tanycytes, investigate their gene regulatory networks, analyze their intercellular communication, and examine their developmental trajectory. Our analysis revealed the presence of two clusters of β tanycytes and three clusters of α tanycytes in our dataset. Surprisingly, human embryonic tanycytes displayed significant similarities to mouse tanycytes in terms of marker gene expression and transcription factor activities. Trajectory analysis indicated that α tanycytes were the first to be generated, giving rise to β tanycytes in a dorsal–ventral direction along the third ventricle. Furthermore, our CellChat analyses demonstrated that tanycytes generated earlier along the developmental lineages exhibited increased intercellular communication compared to those generated later. In summary, we have thoroughly characterized the heterogeneity of human embryonic tanycytes from various angles. We are confident that our findings will serve as a foundation for future research on human tanycytes.

## Introduction

Disruptions in energy homeostasis can lead to diseases such as obesity and diabetes, which affect millions of people every year, compromising their quality of life and even posing life-threatening risks. The tight regulation of energy homeostasis is controlled by the central nervous system, particularly the hypothalamus, which serves as a hub in a complex neural network that meticulously coordinates energy expenditure, food intake, and blood sugar levels^1–3^. The ependymoglial cell, tanycyte, has recently been shown to play critical roles in transmitting information about nutrient sufficiency carried by circulating signals (such as leptin, ghrelin, and insulin) to various hypothalamic neurons, including the orexigenic agouti-related peptide (AGRP) and the anorexigenic proopiomelanocortin (POMC) neurons^4–6^. Tanycytes’ unique location as the floor and side wall of at least part of the third ventricle is instrumental in their function. They have their apical side in direct contact with the cerebrospinal fluid (CSF) from the ventricle and extend their long processes to reach the fenestrated capillaries in the median eminence (ME) and hypothalamic neurons^7^. Furthermore, at least some tanycytes have been shown to also function as adult neural progenitors that can give rise to neurons and astrocytes, contributing to neural plasticity^7^.

Tanycytes are a heterogeneous cell population, consisting of two α subtypes and two β subtypes in the adult rodent hypothalamus^8,9^. These tanycyte subtypes are distributed across different regions along the floor and lateral wall of the third ventricle^8^. Both α and β tanycytes are characterized by their expression of progenitor markers such as Rax and Vim, as well as factors involved in energy balance (Fgf10) and thyroid hormone regulation (Dio2)^8,9^. In mice, Rax exhibits broad expression in both the hypothalamus and retina during the early stages of embryonic development, spanning from embryonic day 7.5 (E7.5) to E13.5. However, its presence becomes progressively limited to tanycytes during the late embryonic stage^10^. Targeted deletion of Rax in the early hypothalamic progenitor cells of mice resulted in a reduction in the expression of genes specific to tanycytes in the wall of the third ventricle, indicating that Rax is essential for the proper differentiation of hypothalamic tanycytes^11,12^. While rodent α tanycytes specifically express Nr2e1, Fabp7, and Vcan, rodent β tanycytes exhibit high expression levels of Col25a1, Aam, as well as Fgf1r, which plays a significant role in glucose homeostasis^8,13^.

So far, most of our knowledge about tanycytes, as presented above, has been acquired in rodents. In humans, tanycytes exhibit a similar distribution as in rodents, and their long processes notably lose organization with age^14^. However, the heterogeneity, regulatory program, intercellular communication and specification trajectory of human tanycytes, among other aspects, remain elusive^15,16^.

The timing of tanycyte emergence during embryonic stages has been observed to vary from one species to another. In rats, the initiation of tanycyte differentiation was noted to begin around E19 (equivalent to E17 in mice) and continued throughout the first two weeks following birth^17^. In contrast, a recent study in mice using single-cell RNA sequencing on Rax+ lineage cells and EdU birthdating has indicated that some radial glia cells transformed into primitive tanycytes as early as E13, with the majority of tanycyte development occurring between E13 and E15^18^. In humans, single-cell studies of the embryonic hypothalamus have observed tanycytes as early as Gestational Week (GW) 6^19^. However, there remains no consensus regarding the hierarchical organization of hypothalamic tanycytes. Some speculate that α tanycytes serve as the primary neural stem cells, while β tanycytes are more specialized neuronal progenitors^7^. This speculation arises from observations that α tanycytes can generate β1 tanycytes and that only α tanycytes exhibit neurosphere-forming abilities^20^. Conversely, another model posits β tanycytes at the top of the hypothalamic neural stem cell hierarchy, with transit-amplifying α tanycytes descending from them^21–23^. However, these studies are primarily based on postnatal or adult mice, prompting the need to elucidate the developmental hierarchy of tanycytes at embryonic stages.

In this study, we gathered and scrutinized embryonic tanycyte cells from two datasets of the human embryonic hypothalamus^19,24^. We clustered these cells, compared them with mouse tanycyte subtypes across various developmental stages, estimated the activities of transcription factors within these human embryonic tanycytes, explored the cell–cell communication between them and other cell types within the hypothalamus, and conducted trajectory analysis to unveil the developmental hierarchy of different embryonic tanycyte subtypes. Our aim was to comprehensively characterize the heterogeneity of human embryonic tanycytes from multiple perspectives, laying the groundwork for future investigations of human tanycytes.

## Materials and methods

### Collect and process human embryonic tanycyte single cell RNAseq data

**Figure 1:**
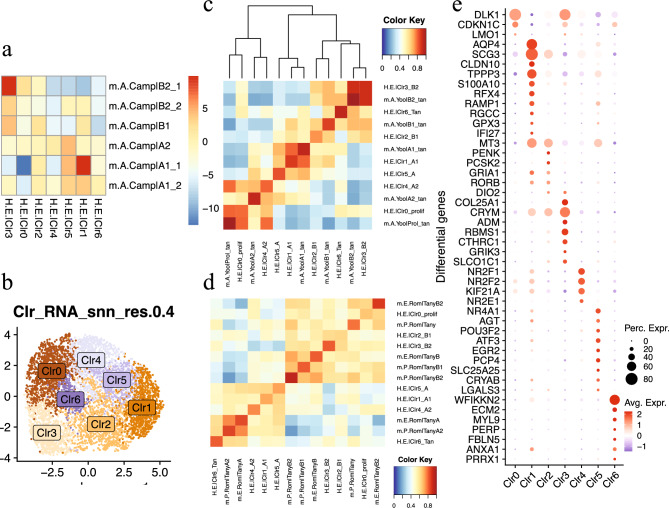
Human embryonic tanycyte clusters: markers and comparison to mouse tanycytes. Panel a illustrates the correspondence between the seven human embryonic tanycyte clusters (i.e. H.E.|Clr*) and the six mouse adult tanycyte subtypes (i.e. m.A.Camp|*) characterized by Campbell et al.^8^, color represents enrichment score. Panel b displays the seven human embryonic tanycyte clusters on UMAP. Panel c depicts the transcriptomic profile similarity between the seven human embryonic tanycyte clusters (i.e. H.E.|Clr*) and five adult mouse tanycyte subtypes characterized by Yoo et al.^23^ (i.e. m.A.Yoo|*). Panel d illustrates the transcriptomic profile similarity between the seven human embryonic tanycyte clusters (i.e. H.E.|Clr*) and three embryonic mouse tanycyte subtypes (i.e. m.E.Rom|*) and four postnatal mouse tanycyte subtypes (i.e. m.P.Rom|*) characterized by Romanov et al.^31^. Panel e shows the top marker gene expression for each of the seven human embryonic tanycyte clusters. In this panel, color indicates the average expression level of a gene within a cluster, while size represents the percentage of cells in the cluster that express the gene.

We obtained a human embryonic hypothalamus dataset from NeMO Analytics (https://nemoanalytics.org/p?l=a856c14e&g=gad2), comprising single-cell RNAseq data from human embryonic hypothalamus generated by both Herb et al.^19^ and Zhou et al.^24^. Tanycytes were isolated from this dataset, which encompassed human developmental gestational weeks (GWs) 6, 7, 8, 10, 12, 15, 16, 18, 19, 20, 22, and 25, with the majority of cells originating from Zhou et al.^24^ (Supplementary Fig. S1_i). Subsequently, the tanycytes underwent reanalysis using Seurat v4.3.0^25^. Before proceeding with further analysis, mitochondrial genes, ribosomal protein genes, hemoglobin genes, sex chromosome genes, and one highly expressed gene, MALAT1 (Supplementary Fig. S2), were removed. Following this the tanycytes were normalized and log-transformed using the NormalizeData function. Next, the CellCycleScoring function was utilized to calculate enrichment scores for cell cycle phases S and G2M by comparing average expression levels across marker gene sets for phases S or G2M with the control expressions. After normalization and log-transformation, all data subsets from each developmental stage of both studies were integrated using Harmony v1.2.0^26^. The integration relied on the differential genes identified for the six mouse adult tanycyte subtypes as characterized by Campbell et al. in 2017^8^. We obtained the differential gene table from Campbell et al.^8^, which conducted pairwise gene differential analyses among all cell types. We selected only those markers that exhibited expression fold-change values of > 1 in at least three of the five pairwise comparisons for each tanycyte subtype. Prior to integration, the normalized counts of those Campbell tanycyte differential genes underwent scaling using the ScaleData function. Simultaneously, we regressed out the total number of genes detected (nFeature_RNA) and the cell cycle scores (S.Score, G2M.Score). Subsequently, the dimensions of the scaled data were reduced using principal component analysis (PCA)^27^. Integration was performed on the top 15 principal components (PCs) from the PCA analysis. Based on the top 15 dimensions of the Harmony reduction, cells were further embedded into a 2-dimensional space using RunUMAP for visualization. To cluster cells into groups, we employed a graph-based clustering approach using the "Louvain" algorithm^28^, which utilized a shared nearest neighbor graph^29^ built on the top 15 dimensions of the Harmony reduction. In total, we identified seven human embryonic tanycyte clusters (Fig. 1_b). Differential genes between the acquired tanycyte clusters were detected using MAST^30^ as implemented in the FindAllMarkers function, with parameters set to max.cells.per.ident = 400 and resolution = 0.4. During the MAST analysis, we controlled for the effect of the number of genes detected (nFeature_RNA), cell cycle S score, and G2M score. Prior to differential gene analysis, all clusters were downsampled to 400 cells.

### Tanycyte heterogeneity: human vs mouse

To discern the tanycyte subtypes represented by the acquired human embryonic tanycyte clusters, we employed a weighted univariate linear model (ULM) using the decoupleR_2.4.0 package ^32^. ULM aims to achieve a similar goal as gene set enrichment analysis, but with each gene in the gene sets weighted based on its contribution to the gene set. For the reference, we utilized the previously mentioned differential gene table of six adult mouse tanycyte subtypes obtained from Campbell et al.^8^, selecting only markers that exhibited expression fold-change values of > 1.5 in at least four of the five pairwise comparisons representing each tanycyte subtype. These marker genes, along with their associated mean gene expression fold change (FC), were utilized to execute ULM on the marker genes (with associated log2FC) identified for each of our tanycyte clusters.

To further check the identities of our tanycyte clusters, we obtained two additional single-cell RNAseq datasets of mouse hypothalamus. One dataset pertained to adult mice (Yoo et al.^23^), while the other encompassed both embryonic and postnatal mice (Romanov et al.^31^). Both studies comprehensively characterized tanycyte subtypes using single cell RNAseq data. With these supplementary mouse single cell RNAseq datasets at our disposal, we performed MetaNeighbor (v1.18.0)^33^ analyses using the MetaNeighborUS function (with fast_version = T) to compare the gene expression profiles of our tanycyte clusters with the tanycyte subtypes identified from these reference datasets.

### Cell–cell communication

To explore the cell–cell communication between tanycyte clusters and other cell types within the hypothalamus, we employed CellChat v1.6.1^34^ to infer biologically significant interactions by assigning each interaction a probability value and conducting a permutation test^34^. CellChat models the probability of cell–cell communication by integrating gene expression data with prior knowledge of interactions between signaling ligands, receptors, and their cofactors using the law of mass action. Subsequently, we computed the communication probability at the signaling pathway level by aggregating the probabilities of all ligand-receptor interactions associated with each pathway. Furthermore, we calculated the aggregate cluster–cluster communication network by tallying the number of links or summarizing the communication probabilities for all ligand-receptor pairs. Additionally, we performed signaling role analysis on the aggregate cluster–cluster communication network to visualize the dominant senders (sources) and receivers (targets) in a 2D space. Finally, we utilized a pattern recognition method to identify global communication patterns, allowing us to visualize how multiple cell groups and signaling pathways coordinate to function.

### Inference of transcription factor activity

The collective interactions of transcription factors (TFs) with their target genes are commonly known as a gene regulatory network (GRN)/regulon, offering a simplified portrayal of the underlying regulatory circuits^35^. In our human embryonic tanycyte dataset, we utilized the ULM method from the decoupleR package to estimate TF activities for each cell, leveraging CollecTRI-derived regulons^36^. To expedite the analysis, we downsampled each tanycyte cluster to a maximum of 300 cells.

For identifying TF markers in each cell type within the human embryonic tanycyte dataset, we employed the FindAllMarkers function (with default settings, except max.cells.per.ident = 300) with a Wilcoxon Rank Sum test from Seurat v5.0.2 on the inferred activity of all TFs. GRNs with an adjusted p-value < 0.05, a log fold change > 0.5, and an activity observed in at least 1% of the cells in a cell type were considered GRN markers for that specific cell type. We conducted a similar analysis (with adjusted settings) on the two aforementioned mouse tanycyte subtype datasets (Yoo et al.^23^, and Romanov et al.^31^). The detected activated regulons for α1, α2, β1, β2 and proliferating tancytes were then integrated together using the aggregateRanks function from the RobustRankAggreg v1.2.1 package^37^.

### Trajectory analysis

To infer cell lineages and chart the developmental progression of human embryonic tanycyte subtypes, We analyzed a combined human embryonic hypothalamus dataset that were obtained by Herb et al.^19^ and Zhou et al.^24^, where each subtype of radial glial cells (RGCs) and other cell types were downsampled to a maximum of 5000 and 1000 cells, respectively. The analysis included filtering, normalization, scaling, dimensional reduction, integration, and UMAP visualization using Seurat as above but with adjusted settings. Subsequently, we isolated tanycytes, ependymal cells, astrocytes, cycling RGC (cRGCs), quiescent RGCs (qRGCs), and supplemented the dataset with our annotations regarding tanycyte subtypes. With this processed dataset, we conducted cell trajectory analysis using the R package Slingshot v2.6.0^38^. Slingshot inferred cell lineages based on the annotated celltypes/subtypes, with the root being cRGC, qRGC. Each celltype/subtype was treated as a distinct cellular state. A branched lineage structure that connects those cellular stages was constructed as a Minimum Spanning Tree (MST)^39^ using cells organized in a UMAP-created reduced-dimensional space.

## Results

### Human embryonic tanycyte heterogeneity

A thorough examination of the tanycyte clusters reveals evident heterogeneity. Utilizing graph-based clustering, human embryonic tanycytes were categorized into seven distinct clusters (Fig. 1_b). Differential gene analysis conducted among these clusters has identified 399 significantly differentially expressed genes (log-fold change > 0.5, adjusted p-value < 0.05; Supplementary Table S1). Notably, within these differentially expressed genes, cluster 1 exhibited significant expression of the α1 tanycyte marker SLC1A2^8^, while cluster 4 showed significant enrichment of the α2 tanycyte marker gene NR2E1^8^ (Fig. 1_e, Supplementary Table S1). Cluster 2 demonstrated significant expression of the β1 tanycyte marker PENK, while cluster 3 exhibited significant enrichment of the β2 tanycyte markers ADM and COLA25A1^8^ (Fig. 1_e). Furthermore, cluster 5 displayed significant expression of the α1 tanycyte marker AGT^8^ (Fig. 1_e). Interestingly, the tanycyte marker CRYM was found to be significantly enriched in clusters 1, 2 & 3 (Supplementary Table S1, Fig. 1_e).

The expression of marker genes overall aligned with our weighted ULM analysis: the most enriched tanycyte subtypes for clusters 3, 0, 2 and 1 were respectively β2 (p-value = 1.6 × 10^–22^), β2 (p-value = 0.02), β1 (p-value = 0.03), and α1 (p-value = 1.2 × 10^–22^) tanycytes (Fig. 1_a, Supplementary Table S2). Additionally, α2 and α1 (p-value < 1.5 × 10^–3^) were nearly equally enriched in cluster 5, while no subtype appeared particularly enriched in clusters 4 and 6 (Fig. 1_a, Supplementary Table S2).

The outcomes of our MetaNeighbour analysis, utilizing Yoo et al.’s adult mouse tanycyte data as a reference^23^, strongly correlated with the results obtained from the analyses of differential gene expression and weighted ULM. For instance, our cluster 3, identified as β2 by marker analysis and weighted ULM, clustered closely with adult mouse β2 tanycytes (m.A.Yoo|B2_tan) from Yoo et al.^23^ in the MetaNeighbour analysis (Fig. 1_c). Similarly, our cluster 2, labeled as β1 through marker analysis and weighted ULM, grouped with Yoo’s β1 tanycytes (m.A.Yoo|B1_tan) in the MetaNeighbour analysis (Fig. 1_c). Furthermore, our clusters 1 and 5, annotated as α1/α tanycytes through marker analysis and weighted ULM, clustered together with Yoo’s α1 tanycytes (m.A.Yoo|A1_tan) in the MetaNeighbour analysis (Fig. 1_c). Our cluster 4, presumed to be α2 tanycyte based on marker gene expression, also clustered with Yoo's α2 tanycyte (m.A.Yoo|A2_tan) in the MetaNeighbour analysis (Fig. 1_c). Notably, our cluster 0 aligned with Yoo's proliferating tanycyte (m.A.Yoo|Prol_tan) (Fig. 1_c), which may also make sense given that cluster 0 comprises predominantly cells from early developmental stages (GW6/7/8/10/12, Supplementary Fig. S1_h) among the seven clusters.

The findings from our MetaNeighbour analysis, referencing Romanov et al.^31^, showed somewhat less alignment with the results of marker gene analysis and weighted ULM. However, all our α tanycytes grouped together with Romanov's α embryonic/postnatal tanycytes, while all our β tanycytes clustered together with Romanov's β embryonic/postnatal tanycytes (Fig. 1_d).

Considering the marker gene expression of our tanycyte clusters and their resemblance to mouse tanycyte subtypes (evaluated through weighted ULM and MetaNeighbour analyses), the most probable subtype identities for our seven clusters are as follows: Cluster 0: proliferating, Cluster 1: α1, Cluster 2: β1, Cluster 3: β2, Cluster 4: α2, Cluster 5: α, Cluster 6: tanycytes.

### Transcription factor activities

The control of gene expression at the transcriptional level involves transcription factors (TF) that either activate or inhibit transcription. In this study, we evaluated the activities of transcription factors in human embryonic tanycytes and computed activity scores for 764 regulons using decoupleR. The activity scores for these regulons are provided in Supplementary Table S3.

In addition, we computed regulon activity scores for Yoo's adult mouse tanycytes and Romanov's embryonic/postnatal tanycytes for comparison (Supplementary Table S4 & S5). Surprisingly, human embryonic tanycytes exhibited a high degree of similarity to mouse tanycytes in terms of the activities of transcription factors (TFs) crucial for astroglial and tanycyte cell specification (Supplementary Fig. S3). For instance, both mouse and human tanycytes demonstrated elevated activities of NFIA/Nfia, EZH2/Ezh2, E2F1/E2f1, NR1D1/Nr1d1, MAF/Maf, and TBX3/Tbx3—TFs pivotal for tanycyte specification^31^. Notably, Nfia-knockout mice showed impaired formation of tanycytes and astrocytes at E18.5^31^.

Furthermore, both human embryonic tanycytes and mouse embryonic/postnatal mice exhibited high activity of SOX9, whereas this activity was not observed in mouse adult tanycytes.

**Figure 2:**
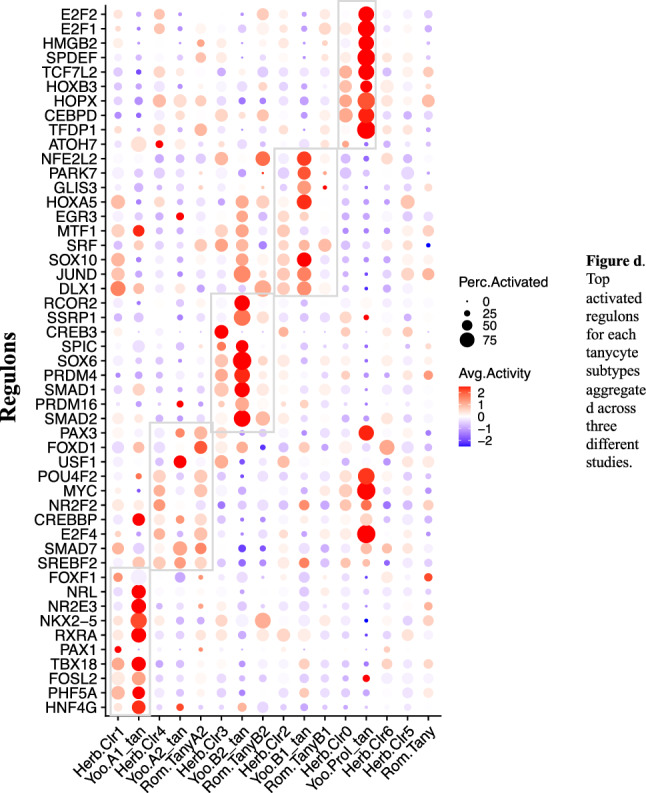
Top activated regulons for each tanycyte subtypes aggregated across three different human tanycyte studies. Herb.Clr* represents the seven tanycyte clusters identified in the current study, utilizing data downloaded from Herb et al.^19^, which also includes tanycyte data collected by Zhou et al.^24^; Yoo.* refers to tanycyte clusters identified by Yoo et al.^23^, while Rom.* designates tanycyte clusters detected by Romanov et al.^31^. In this plot, color indicates the average activity of a gene within a cluster, while size represents the percentage of cells with the regulon activated within that cluster. Regulon markers of similar clusters are enclosed in rectangles.

At the tanycyte subtype level, we have identified distinct regulons for each cluster of human embryonic tanycytes, as well as for each Yoo’s and Romanov’s tanycyte subtype (Supplementary Tables S6, S7 & S8 and Supplementary Fig. S4). To determine consensus activated transcription factors (TFs) for each subtype, we aggregated ranked lists of differential regulons (based on average log2 fold change) from different datasets for α1, α2, β2, β21 and proliferating tanycytes (Supplementary Tables S9, S10, S11, S12, S13). We then plotted the activity scores of the top activated regulons for these subtypes together, revealing that the top activated regulons for α1 tanycytes included PHF5A/Phf5a, FOSL2/Fosl2, TBX18/Tbx18, and RXRA/Rxra, while the top activated regulons for β2 tanycytes included SMAD1/Smad1, PRDM4/Prdm4, SOX6/Sox6, and SPIC/Spic (Fig. 2). Among those activated regulons, SMAD1 mediates the signals of bone morphogenetic proteins (BMPs), which play a role in various biological activities, including gliogenesis and neurogenesis^40,41^.

### Cellular communication

In total, CellChat identified 4654 significant ligand–receptor pairs that were differentially overexpressed for human embryonic tanycyte clusters, and those ligand-receptor pairs were further categorized into 42 signaling pathways. The significant signaling pathways and ligand-receptor pairs are detailed in Supplementary Table S14. Additionally, we computed signaling network interaction counts and interaction weights, which are provided in Supplementary Tables S15 and S16, respectively.

Overall, β2 tanycytes demonstrated slightly lower communication levels compared to α tanycytes (Supplementary Fig. S5_d). Additionally, cluster 3 of β2 tanycytes exhibited the lowest level of incoming cell–cell communication, while clusters Clusters 0 (proliferating), 4 (α2), and 6 demonstrated fewer outgoing interactions compared to clusters Clusters 1 (α1), 2 (β1), and 5 (α) (Supplementary Fig. S5_e). Moreover, all tanycyte clusters displayed slightly stronger connections with astrocytes, oligodendrocytes, qRGC, and IntProgen_1 (one intermediate progenitor cluster) compared to other cell types (Supplementary Fig. S6_d). Furthermore, the two β tanycyte clusters (Clusters 2 & 3) exhibited increased intercellular interactions with endothelial cells (Supplementary Fig. S6_c). Notably, a unique ligand-receptor pair, ADM-CALCRL, was identified between β2 Cluster 3 and endothelial cells, with only Cluster 3 acting as the receiving partner (Supplementary Fig. S5_a). ADM is a marker gene of β2 tanycytes. Another noteworthy interaction involving β2 Cluster 3 is MPZL1-MPZL1 (Supplementary Fig. S5_b), through which β2 Cluster 3 communicated with oligodendrocytes, endothelial cells and VLMC.

**Figure 3:**
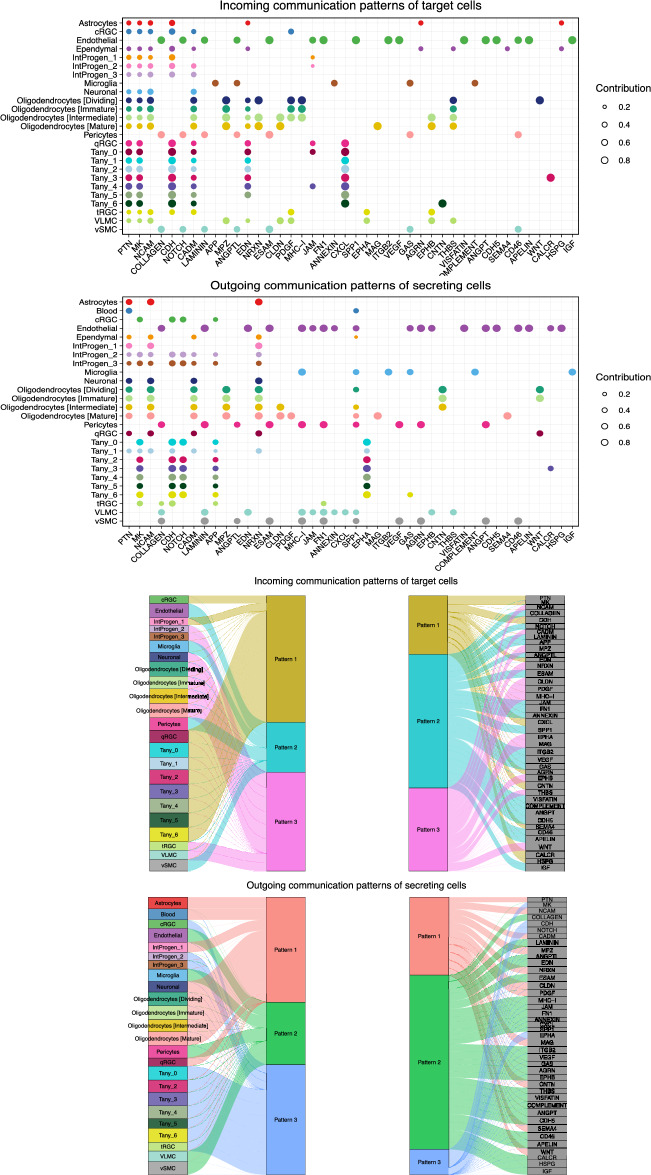
The inferred incoming and outgoing communication patterns of hypothalamus cells. Incoming patterns illustrate how target cells (i.e., cells acting as signal receivers) coordinate among themselves and with specific signalling pathways to respond to incoming signals. Outgoing patterns reveal how sender cells (i.e., cells acting as signal sources) coordinate among themselves and with specific signalling pathways to facilitate communication. In essence, cell communication patterns are categorized based on the similarity of their communication processes. (**a**) Dot plot displaying the incoming communication patterns of target cells. The y-axis shows all cell types identified by Herb et al.^19^, as well as the seven tanycyte clusters identified in the current study, while x-axis shows various signalling pathways. Dot size represents the contribution of each cell type to a particular pathway. (**b**) Dot plot displaying outgoing communication patterns of secreting cells. (**c**) Inferred incoming communication patterns of target cells, showcasing how these cells coordinate with each other and with specific signalling pathways to respond to signals. The left column displays the cell types, the two middle columns present the signalling patterns, and the right column shows the signalling pathways. (**d**) Inferred outgoing communication patterns of secreting cells, illustrating the relationship between the inferred latent patterns, cell groups, and signalling pathways. The thickness of the flow indicates the contribution of the cell group or signalling pathway to each latent pattern.

In addition, a significant inquiry involves understanding how multiple cell groups and signaling pathways synchronize their functions. To address this question, CellChat employed a pattern recognition method based on non-negative matrix factorization to identify the global communication patterns and key signals within different cell groups. Our investigation revealed three distinct patterns for both incoming and outgoing signaling. The communication patterns of target cells indicated that the incoming signaling of all tanycyte subtypes was primarily influenced by pattern 1, encompassing signaling pathways such as CALCR, CDH, and PTN. Conversely, the outgoing signaling of all tanycyte subtypes was characterized by pattern 3, which included pathways like MK, CDH, NOTCH, and EPHA (Fig. 3).

### Different specification trajectories for the β and α embryonic tanycytes

**Figure 4:**
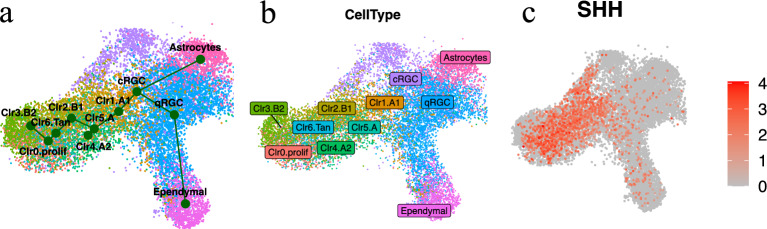
Slingshot trajectory of human embryonic tanycytes. (**a**) The inferred trajectory encompasses all human embryonic tanycytes studied here, alongside two populations of radial glial cells (qRGC and cRGC), astrocytes, and ependymal cells. (**b**) Cell type distribution on UMAP. (**c**) The cellular expression levels of SHH visualized on UMAP.

The Slingshot trajectory analysis has identified two specification lineages among the embryonic tanycyte cells. One lineage originates from the quiescent radial glial cell population qRGC and passes through the cycling radial glial cell population cRGC, α1 tanycyte cluster 1, α tanycyte cluster 5, eventually leading to α2 tanycyte cluster 4. The second lineage also begins from qRGC and traverses through cRGCs, α1 tanycyte cluster 1, α tanycyte cluster 5, β1 tanycyte cluster 2, cluster 6, the proliferating tanycyte cluster 0, ultimately reaching β2 tanycyte cluster 3 (Fig. 4).

## Discussion

In mammals, adult hypothalamic stem cells, called tanycytes, are pivotal in aiding hypothalamic neurons to uphold metabolic balance. This study delves into human embryonic tanycytes that were generated by two single-cell transcriptomics studies^19,24^, unveiling their diversity, comparing them to mouse adult/postnatal/embryonic tanycytes, scrutinizing their transcription factor activities, examining their intercellular communication, and elucidating their developmental trajectories.

### Human tanycytes are very similar to mouse tanycytes

In summary, we have identified three populations of α, two of β, and one proliferating tanycytes, all of which were present as early as GW6, the earliest time point in our dataset, suggesting their onset during early brain development (Supplementary Fig. S1_h&i). Exploring developmental stages before GW6 may further reveal the earlier developmental program of tanycytes in humans. Surprisingly, our tanycytes exhibited a high degree of similarity to mouse adult/postnatal/embryonic tanycytes in both transcriptomic profiles and transcription factor (TF) activities (Fig. 1 and Supplementary Fig. S3). Whether human or mouse, embryonic, postnatal, or adult, all tanycytes required NFIA/Nfia, EZH2/Ezh2, E2F1/E2f1, and NR1D1/Nr1d1 to be activated, suggesting their fundamental role in tanycyte functionality and development. Notably research on Nfia-knockout mice has revealed impaired development of tanycytes and astrocytes at E18.5^31^. Furthermore, on postnatal mice, targeted disruption of the NFI family of transcription factors (Nfia/b/x) specific to tanycytes strongly induced tanycyte proliferation and neurogenesis originating from tanycytes^23^. In contrast, only embryonic and postnatal tanycytes necessitated activation of SOX9, while adult tanycytes did not. This indicates the importance of SOX9 regulon maybe only at fate specification stages.

### Tanycyte developmental trajectory: radial glia cells (RGCs)—α tanycytes—β tanycytes

In the hypothalamus, the four classical tanycytes subtypes (α1, α2, β1, and β2) are distinguished by their dorsoventral positioning along the third ventricle. β2 tanycytes are situated at the base of the third ventricle, while β1 tanycytes start from the periphery of the ventricle floor and extend slightly upwards along its lateral wall. α2 tanycytes are positioned just above β1 tanycytes on the lateral wall. In contrast, α1 tanycytes occupy the most dorsal position among the four subtypes^22^.

Our trajectory analyses indicated that tanycyte development followed a dorsal–ventral direction along the third ventricle: α tanycytes, particularly α1, initially originated from RGCs and subsequently gave rise to β tanycytes, with β2 tanycytes at the tip (Fig. 4). Similarly, in adult mice, α tanycyte cells have been shown to consistently generate other cell types, including β1 tanycytes^20^. Furthermore, tanycytes composing the lateral walls of the ventricle (β1 and α tanycytes) are known to develop from Shh-expressing floor-plate/ventrolateral cells^42^. This finding may be consistent with our results: cycling RGCs and some quiescent RGCs expressed SHH, and it's noteworthy that all tanycytes, except those at or near the lineage tips (e.g. β2 tanycyte), also exhibited high levels of SHH expression (Fig. 4).

### α1 tanycytes communicate more than β2 tanycytes

Signaling interactions involving soluble and membrane-bound factors play a crucial role in cellular functions and development^34^. In this study, utilizing CellChat analysis, we noted that tanycyte subtypes situated at early stages of the developmental trajectory (α1 cluster 1, α cluster 5, β1 cluster 2, and α2 cluster 4) exhibited more extensive communication with other cells compared to those appearing later along the developmental lineages (cluster 6, proliferating cluster 0, and β2 cluster 3) (Supplementary Fig. S5_d&e). Similarly, we observed elevated expression of the GJA1 gene among α1 tanycyte clusters (Supplementary Fig. S5_c). GJA1 serves as a component of gap junctions, facilitating intercellular communication between adjacent cells^43^. These findings suggest that cells at early developmental stages along the lineages necessitate coordination within a more complex intercellular network. For β2 tanycytes situated at the apex of the second lineage, two specific signaling pathways are particularly interesting: CALCR and MPZ. Each pathway comprises only one ligand-receptor pair, namely ADM-CALCRL and MPZL1-MPZL1, respectively and both interactions involve endothelial cells (Supplementary Fig. S5_a&b). ADM potentially functions as a hormone in the regulation of circulation^44^, requiring interaction with two receptors, CALCRL and RAMPs, both of which are expressed in β tanycyte clusters (Supplementary Fig. S5_c)^45,46^. MPZL1 (PZR) is a glycoprotein involved in extracellular matrix-induced signal transduction^47,48^. These findings align with the role of β2 tanycytes as constituents of the blood–brain interface, which dynamically regulate the passage of nutrients and hormones to the brain^2,7^.

### Supplementary Information

Supplementary Figures.

Supplementary Table 1.

Supplementary Table 2.

Supplementary Table 3.

Supplementary Table 4.

Supplementary Table 5.

Supplementary Table 6.

Supplementary Table 7.

Supplementary Table 8.

Supplementary Table 9.

Supplementary Table 10.

Supplementary Table 11.

Supplementary Table 12.

Supplementary Table 13.

Supplementary Table 14.

Supplementary Table 15.

Supplementary Table 16.

## Data Availability

All data generated during this study are included in this published article [and its supplementary information files].
