# Supplementary material for: A single-cell transcriptomic study of heterogeneity in human embryonic tanycytes
Source: Sci Rep. 2024 Jul 4;14:15384. doi: 10.1038/s41598-024-66044-7 (PMC11224400; doi:10.1038/s41598-024-66044-7)
Supplement: Supplementary file 1 — Supplementary Figures. [file 41598_2024_66044_MOESM1_ESM.docx]

**Supplementary Table titles**

**Supplementary Table S1**. Differential gene table for the seven human embryonic tanycyte clusters.

**Supplementary Table S2**. Enrichment table for the seven human embryonic tanycyte clusters in the adult mouse tanycyte subtypes identified by Yoo et al (2021) [1].

**Supplelementary Table S3**. Regulon activity scores for 764 regulons within each human embryonic tanycyte cell.

**Supplelementary Table S4**. Regulon activity scores for 764 regulons within each mouse adult tanycyte cell characterized by Yoo et al. (2021) [1].

**Supplelementary Table S5**. Regulon activity scores for 764 regulons within each mouse embryonic/postnatal tanycyte cell characterized by Romanov et al. (2020) [2].

**Supplelementary Table S6**. Differential Regulon table for human embryonic tanycyte clusters. GRN: gene regulatory network.

**Supplelementary Table S7**. Differential Regulon table for mouse adult tanycyte subtypes characterized by Yoo et al. (2021) [1]. GRN: gene regulatory network.

**Supplelementary Table S8**. Differential Regulon table for mouse embryonic/postnatal subtypes characterized by Romanov et al. (2020) [2]. GRN: gene regulatory network.

**Supplelementary Table S9**. Aggregated regulon list ranked by Aggregation scores/p value for α1 tanycytes.

**Supplelementary Table S10**. Aggregated regulon list ranked by Aggregation scores/p value for α2 tanycytes.

**Supplelementary Table S11**. Aggregated regulon list ranked by Aggregation scores/p value for β2 tanycytes.

**Supplelementary Table S12**. Aggregated regulon list ranked by Aggregation scores/p value for β1 tanycytes.

**Supplelementary Table S13**. Aggregated regulon list ranked by Aggregation scores/p value for prolifering tanycytes.

**Supplementary Table S14**. Significant ligand-receptor pairs and associated signaling pathways as detected in human embryonic hypothalamus (including tanycyte clusters).

**Supplementary Table S15**. Counts of signaling network interactions for human embryonic hypothalamus (including tanycyte clusters).

**Supplementary Table S16**. Weights of signaling network interactions for human embryonic hypothalamus (including tanycyte clusters).

**Supplementary Figures**

**Supplementary Figure S1.** Basic information regarding the human embryonic tanycyte dataset. Panels a, b, c, and d depict the distributions of ages/studies, individual studies, cell cycle phases, and ages on UMAP, respectively. Panels e and f display the levels of cell cycle S score and G2M score for each tanycyte cluster. Panel g illustrates the proportion of cells at different cell cycle phases for each tanycyte cluster, while panel h shows the proportions of cells at different ages for each cluster. Lastly, panel i presents the cell count of the two studies at different developmental stages.

**Supplementary Figure S2**. Top highly expressed genes in the human embryonic tanycyte dataset.

**Supplementary Figure S3.** Activity UMAP plots for transcription factors that are important for the specification of astroglial cells, tanycytes and neurons in Human embryonic tanycytes identified by the current study (a), adult mouse tanycytes identified by Yoo et al. (2021) [1] (b), embryonic tanycytes identified by Romanov et al. (2020) [2] (c), as well as postnatal tanycytes identified by Romanov et al. (2020) [2] (d).

**Supplementary Figure S4.** Top activated regulons for different subtypes of human embryonic tanycytes identified by the current study (a), mouse adult tanycytes identified by Yoo et al. (2021) [1] (b), mouse embryonic tanycytes identified by Romanov et al. (2020) [2] (c) and mouse postnatal tanycytes identified by Romanov et al. (2020) [2] (d). GRN: gene regulatory network. In those plots, color indicates the average activity of a gene within a cluster, while size represents the percentage of cells with the regulon activated within that cluster.

**Supplementary Figure S5.** Intercelluar communication network related information. Panels **a** and **b** display circle plots depicting the inferred intercellular communication network for ligand pairs ADM-CALCRL and MPZL1-MPZL1. The thickness of the lines represents the number of interactions and the strength of interaction weights between two cell types. Panel **c** showcases feature plots of several significant or interesting ligands/receptors. Panel **d** provides information on the total number of intercellular interactions and the summarized intercellular interaction weights, respectively. Panel **e** offers an overview of the summarized strengths of both incoming and outgoing intercellular interactions.

**Supplementary Figure S6**. Interaction plot of hypothalamus cell types. Panel a illustrates the count of intercellular interactions, while Panel b depicts the interaction weights. Panel c displays the count of interactions originating from each of the seven human embryonic tanycyte clusters identified in the current study, while Panel d shows the weight of the interactions originating from each of these clusters. Thicker lines indicate a higher number of interactions or stronger interaction weights/strength between two cell types.

1. Yoo, S. et al. Control of neurogenic competence in mammalian hypothalamic tanycytes. *Sci. Adv.* **7**, eabg3777 (2021).
2. Romanov, R. A. et al. Molecular design of hypothalamus development. *Nature*. **582**, 246-252 (2020).
